# Preventing socioeconomic inequalities in health behaviour in adolescents in Europe: Background, design and methods of project TEENAGE

**DOI:** 10.1186/1471-2458-9-125

**Published:** 2009-05-08

**Authors:** Frank J van Lenthe, Ilse de Bourdeaudhuij, Knut-Inge Klepp, Nanna Lien, Laurence Moore, Fabrizio Faggiano, Anton E Kunst, Johan P Mackenbach

**Affiliations:** 1 Department of Public Health, Erasmus University Medical Center Rotterdam, Rotterdam, the Netherlands; 2 Department of Movement and Sport Sciences, Faculty of Medicine and Health Sciences, Ghent University, Ghent, Belgium; 3 Department of Nutrition, Faculty of Medicine, University of Oslo, Oslo, Norway; 4 Cardiff Institute of Society, Health and Ethics, Cardiff University, Cardiff, UK; 5 Department of Clinical and Experimental Medicine, Avogadro University, Novara, Italy

## Abstract

**Background:**

Higher prevalence rates of unhealthy behaviours among lower socioeconomic groups contribute substantially to socioeconomic inequalities in health in adults. Preventing the development of these inequalities in unhealthy behaviours early in life is an important strategy to tackle socioeconomic inequalities in health. Little is known however, about health promotion strategies particularly effective in lower socioeconomic groups in youth. It is the purpose of project TEENAGE to improve knowledge on the prevention of socioeconomic inequalities in physical activity, diet, smoking and alcohol consumption among adolescents in Europe. This paper describes the background, design and methods to be used in the project.

**Methods/design:**

Through a systematic literature search, existing interventions aimed at promoting physical activity, a healthy diet, preventing the uptake of smoking or alcohol, and evaluated in the general adolescent population in Europe will be identified. Studies in which indicators of socioeconomic position are included will be reanalysed by socioeconomic position. Results of such stratified analyses will be summarised by type of behaviour, across behaviours by type of intervention (health education, environmental interventions and policies) and by setting (individual, household, school, and neighbourhood). In addition, the degree to which effective interventions can be transferred to other European countries will be assessed.

**Discussion:**

Although it is sometimes assumed that some health promotion strategies may be particularly effective in higher socioeconomic groups, thereby increasing socioeconomic inequalities in health-related behaviour, there is little knowledge about differential effects of health promotion across socioeconomic groups. Synthesizing stratified analyses of a number of interventions conducted in the general adolescent population may offer an efficient guidance for the development of strategies and interventions to prevent socioeconomic inequalities in health early in life.

## Background

Throughout Europe, there are robust and persistent socioeconomic inequalities in health. The reduction of these inequalities is among the major priorities of public health policy in (countries of) Europe. Studies conducted among adults have shown that unhealthy behaviours – physical inactivity, unhealthy eating habits, smoking, and alcohol consumption – are more prevalent in lower socioeconomic groups, and contribute to socio-economic inequalities in health [1-5]. Health promotion efforts that are particularly effective in lower socioeconomic groups therefore offer an important route for bridging the gap in health between higher and lower socioeconomic groups. Strategies to tackle inequalities in health-related behaviour are thus far mainly concentrated on the *reduction *of inequalities in unhealthy behaviours among adults [6], for example through community-based approaches [7] and/or through specific efforts to better reach lower socioeconomic groups with evidence-based interventions [8]. Currently, less is known about the *prevention *of socioeconomic health inequalities. During the life-course socioeconomic inequalities in unhealthy behaviours must develop and early periods in the life-course logically offer the ideal opportunity for preventing the development of socioeconomic inequalities in unhealthy behaviour and ultimately health.

For health promotion, adolescence is an important period in life. It is the period where individuals initiate smoking and start consuming alcohol [9]. Moreover, adolescence is characterized by marked declines in physical activity [10,11] and an increase in unhealthy dietary habits, such as the intake of sugar-sweetened beverages [12] Together, these behaviours have resulted in increasing prevalence rates of overweight and obesity. Longitudinal studies have shown that these behaviours to some extent track into adulthood [13,14], and associations have been reported with mortality in middle age adulthood [15,16]. Thus, effective prevention of unhealthy behaviour or promotion of healthy behaviour in adolescence is of crucial importance.

There are good reasons to expect an inverse association between SEP and unhealthy behaviours in adolescence: established and well-described socioeconomic inequalities in unhealthy behaviours in adulthood most likely have their origin earlier in life. However, it is suggested that socio-economic inequalities in unhealthy behaviours already apparent in childhood "equalize" in adolescence, as a results of a declining influence of parents and an increasing influence of significant others (e.g. peers) [17]. For some health outcomes, it can even be speculated that a higher prevalence of unfavourable health indicators is more frequent among adolescents in higher socioeconomic groups, for example when unhealthy behaviours are a reaction to the higher perceived stress due to expected achievements.

Empirical evidence, summarized in a review of the literature, mainly points towards inverse associations between SEP and unhealthy behaviours [18]. A majority of studies reviewed found an inverse association between SEP and smoking in adolescence, the relationship being more consistent in adolescents in early than in later adolescence. Apparently, adolescents in lower socioeconomic groups start smoking at younger ages. Similarly, a majority of reviewed studies showed an inverse association between SEP and inadequate diets, with lower consumption of fruits and vegetables, and higher intake of (refined) fat. A similar association was also found for physical activity, with a majority of studies showing greater physical activity among those in higher socioeconomic groups, perhaps particularly in older adolescents and in girls. There is some evidence that socioeconomic inequalities in overweight, being the results of a combination of socioeconomic inequalities in physical activity and diet, have increased over time [19]. The exception is the absence of an association between SEP and alcohol consumption in adolescence.

These studies provide a clear rationale to develop interventions and policies aimed at the reduction of socioeconomic inequalities in health-related behaviours among adolescents. Although it is sometimes assumed that some health promotion strategies may be particularly effective in higher socioeconomic groups, thereby increasing socioeconomic inequalities in health-related behaviour, actually there is little knowledge about the differential effects of health promotion across socioeconomic groups.

In the past years, a large number of studies have been conducted with the aim of investigating the effectiveness of interventions to improve health-related behaviour in adolescents. Given the potential confounding effect of SEP, many studies have incorporated and statistically adjusted analyses for indicators of SEP. These studies provide a pragmatic opportunity to investigate the differential effectiveness of interventions across socioeconomic groups. Re-analyses of existing interventions stratified by SEP is a relatively quick and cost-effective approach, through which we can learn which interventions best contribute to the prevention of socioeconomic inequalities in health-related behaviours in adolescents.

It is the aim of project TEENAGE to re-analyze a selection of intervention studies conducted in Europe, and to assess the transferability of these interventions across Europe. This paper outlines the design and methods used in the TEENAGE project.

## Methods/design

### Selection of intervention studies

In TEENAGE, interventions are included concerning four health-related behaviours: physical activity, diet, smoking, and alcohol consumption. A coordinated and systematic search strategy in the PubMed database identifies eligible intervention studies conducted in Europe. Table 1 provides the inclusion criteria for interventions eligible to be included in TEENAGE. Studies conducted before 1990 are excluded in the project, as the societal context, the prevalence of the behaviours and their underlying determinants may be different from the current context. Details of the search strategy are described in appendix 1.

**Table 1:** Selection criteria for inclusion of interventions in the search strategy for selection of interventions in Teenage

- Intervention studies which appeared in the international scientific literature since 1995, and conducted since 1990 are included; studies published before 1995 and/or interventions conducted before 1990 are excluded from the search
- Only studies published in the scientific literature are included; grey literature is excluded from the search
- Only studies written/published in English-language are included
- Only studies conducted in Europe are included.

The search strategy results in a list of potentially relevant publications. For each of the four behaviours, one person reads all the titles of the studies, and excludes those that – based on the titles – are not relevant. In case of doubt, the study will remain in the list. In the next step, abstracts are read of all remaining papers and based on the abstracts studies are excluded that are not relevant for the purpose of the project. At this stage, it is important that studies conducted an evaluation (at least a pre-post measurement) and provided a brief description of what was evaluated. Although it is important that studies ultimately selected should have measured an indicator of SEP, they are not excluded if nothing is mentioned in the abstract about an indicator of SEP. From the remaining papers, the full text is retrieved and one person extracts all relevant information. For this purpose, a database is constructed in which all information can be entered.

All identified studies are entered in the TEENAGE intervention database. Based on this information, a final selection of interventions is made, a process in which considerations about the inclusion of different types of interventions and a priori expectations about effectiveness in lower socioeconomic groups is important (please see below). After the final selection, project leaders of the studies selected for the proposed re-analysis are invited to participate in TEENAGE.

### Which health promotion interventions are potentially relevant?

In the project, three broad types of interventions are distinguished: 1) health education, 2) environmental interventions and 3) policies and laws. Based on available knowledge on the explanation of socioeconomic inequalities in health-related behaviours, some priorities are formulated for potentially relevant interventions.

#### Health education

Health education can be defined as "planned learning experiences to facilitate voluntary change in behaviour" [20]. Initially, a main purpose of health education concerned the transmission of information and it therefore seems straightforward to use health education for the promotion of health in lower educational groups. This could work to the extent that these inequalities are the result of a lack of information. While there may be complex health issues for which differences in knowledge between higher and lower educated groups may be an important mechanism [21,22], this seems to be a marginal explanation for socioeconomic inequalities in the behaviours include in TEENAGE. Other aspects of health education include skills training, social influences approaches and motivational interviewing [23]. For example, important skills for adolescents in the context of smoking and alcohol consumption may concern dealing with peer pressure. It remains less clear to what extent differential motivation across socioeconomic groups may explain socioeconomic inequalities in health-related behaviours. It is known in adults that there seems to be no socioeconomic inequalities in the intention to quit smoking [24], but successful quitting is more complicated as a result of the circumstances in which individuals live [25,26].

The latter issue refers to an important potential limitation of health education for the purpose of reducing socioeconomic inequalities in health and health-related behaviours: it does not take into account the circumstances in which individuals live. According to socio-ecological theories, health behaviours should be understood in their context [27,28] and this context may substantially differ for higher and lower socio-economic groups. A major innovation in health education in the past decade concerns the development of tailored interventions [29]. These tailored interventions seem to be better equipped to include specific determinants of health behaviour and to provide tailored feedback taking into account the individual's context. In TEENAGE, an emphasis will therefore be given to the inclusion of tailored interventions of health promotion.

Recognizing the potential importance of the environment, the focus on health education has shifted towards 'health promotion', which can be defined as "the combination of educational and environmental supports for actions of living conducive of health" [20] Health promotion thus includes the above mentioned interventions, as well as interventions in the environment and policies and laws.

#### Environmental interventions

Because of the collective nature of unhealthy behaviour in lower socioeconomic groups, it is reasonable to hypothesize that these inequalities are rooted in unfavourable environmental circumstances shared by individuals in lower socioeconomic groups. Differences in the social environment of adolescents (e.g. in unhealthy behaviours of peers, siblings and parents) may contribute to socioeconomic inequalities in these behaviours. It is also thought that there is a differential exposure to health-related facilities in the physical environment for youth living in socioeconomic different neighbourhoods. Observational research in adults on the explanation of socioeconomic inequalities in health shows that environmental characteristic mediate the association between SEP and health-related behaviours [30-33]. Therefore, there will be special emphasis to include interventions aimed at changing elements of the environment in TEENAGE.

#### Policies and laws

Policies and laws generally apply to everybody for whom they have been made: School policies are the same for all students and governmental laws apply to all residents of the country. At the national level, laws may target prices of cigarettes and alcohol, abandon advertising for cigarettes and alcohol, and restrict sales to adults. At the school level, policies may be adopted to increases physical education or to provide fruits for free. Particularly for smoking, evidence suggests that policies and laws are effective in reducing socioeconomic inequalities in smoking [34,35] Consequently, in the selection of interventions, it will be specifically searched for studies evaluating policies and laws.

#### Settings

Additional information by which interventions will be classified in the project include the setting in which the intervention was conducted. A distinction will be made in interventions targeting the individual, and those conducted in the setting of the household, the school, and the wider community. Thus, this classification will not be used as a criterion to in- or exclude studies, but will serve the purpose of concluding on the most appropriate settings for interventions aimed at reducing inequalities in unhealthy behaviours.

In order to classify the environmental interventions, policies and laws the ANGELO (ANalysis Grid for Environments Linked to Obesity) framework will be applied [36]. This framework is originally constructed to classify environmental determinants of obesity by type of environment (physical, socio-cultural, economic and political) and by setting (micro: home, school, neighbourhoods/friends and macro: national). Table 2 provides the framework, along with potential interventions in cells of the framework.

**Table 2:** Settings for and types of health promotion in TEENAGE, including some examples of potential interventions in cells of the table.

Setting	Health education	Environmental interventions			
	Individual	Physical	Socio-cultural	Economical	Political
**Individual**	- mass media				
	- brochures, leaflets				
	- tailored interventions				
					
**Micro**					
					
Home		- availability cigarettes, alcohol, exercise equipment, fruit	- provision family support		- targeting parenting style
					
School	- school-based health education	- availability healthy and unhealthy canteen food, exercise equipment	- targeting peer pressure	- changing costs of healthy and unhealthy food	- changing school policies with regard to health promotion
				- providing free breakfast in schools	- increasing numbers of hours of physical activity
					
Neighbourhood/friends	- bill boards	- availability shops selling cigarettes, alcohol, fruit, vegetables, exercise places, parks	- peer pressures	- prices of exercise facilities	
					
		*Laws and policies*			
					
**Macro**					
					
National		- opening hours bars		- taxation	- bans on advertisement

### Measures in intervention studies

#### Indicators of socioeconomic position in adolescence

Studies in TEENAGE will be stratified by indicators of SEP and it is therefore crucial to reflect on the measurement of indicators of SEP in adolescence. As mentioned, studies identified will most likely have included an indicator of SEP to be used as a confounding variable in analyses, and consequently, it cannot be excluded that less attention has been paid to the selection of the most appropriate indicator of SEP. In TEENAGE, different indicators are allowed to be included in the intervention studies and there will be no selection based on the type of the indicator. However, for the interpretation of the results, some issues will need to be taken into account.

In adults, three core indicators of SEP are education, income and occupation. Adolescents however, often will not have finished their education already, and they do not have an occupation or full income. To reflect SEP in adolescence, parental indicators of education, occupation and income are appropriate, but considerations need to be acknowledged. Firstly, these three core indicators may reflect different aspects of SEP; while education often is regarded to reflect knowledge, occupation may reflect social standing and income seems to reflect access to material resources [37]. Secondly, the indicators may be either measured for the mother or the father or for both. Although it can not a priori be decided which indicator will be more strongly associated with health behaviours in adolescence, it is important to recognize that associations may differ if using an SEP indicator for the mother or the father. Thirdly, there are indications that adolescents are able to validly report parental occupation and education, but misclassification and the occurrence of missing values can be reasonably expected [38,39]. To the extent that such problems occur more frequently in lower SEP groups, socioeconomic inequalities in health-related behaviours may be underestimated. To avoid these methodological problems, Currie *et al *have proposed to use the family affluence scale (FAS) in the Health Behaviour of School-aged Children Survey (HBSC) [40]. The FAS includes questions on the number of phones and cars in the household, family holiday frequency and whether the child owns an unshared bedroom; high response rates have been achieved [40].

The above-mentioned indicators are all derived from socioeconomic characteristics of parents. Can we use information from adolescents themselves? Adopting a life-course perspective for the development of socioeconomic inequalities in health-related behaviour would allow reasoning that information about levels of education on adolescents predict their entrance position on the labour market, their future income and their lifestyle in adulthood. Indeed, the (additional) inclusion of indicators of education in adolescence has been advocated [41]. In the latter study, adolescent's personal and social position was based on schooling. Students were asked to rate whether they believed to be average, above or below average as compare to schoolmates. Studies have reported important associations between indicators obtained from adolescents and health [41], justifying the inclusion of such indicators as well.

#### Health-related variables

Total physical activity is the sum of many activities, including exercise at school, sports activities, other leisure time activities and transport. All interventions aimed at promoting these forms of activity are eligible for inclusion in the project. Interventions aimed at reducing sedentary behaviour are not included in TEENAGE.

An unhealthy diet in adolescence mainly concerns low fruit and vegetable intake, a high intake from sugar-sweetened soft drinks, a high fat intake and high snack consumption. In early adolescence, the prevalence of these unhealthy behaviours is already substantial. Interventions included in TEENAGE will therefore concentrate on preventive efforts aimed at preventing fat intake and snack consumption, and promoting fruit and vegetable intake.

The substantial majority of adult smokers report that they began smoking in their teenage years. Prevalence of weekly smoking increases dramatically from 5% among 12–13 year olds to levels ranging by country from 8–32% by age 15 [9]. While adult smoking rates have come down over recent years, this has largely been due to cessation in adults rather than in any substantial progress in reducing smoking rates among older adolescents, particularly among girls. Thus, studies will be searched for in which interventions aimed at the prevention of smoking are evaluated; smoking cessation interventions will not be included in the search.

In early adolescence, the prevalence of alcohol consumption will also be low. Interventions in early adolescence will therefore aim at preventing the uptake of alcohol consumption. In later adolescence, the emphasis may shift to preventing large amounts of alcohol consumption, including so-called binge-drinking, which will also be included in the study.

### Statistical methods

In intervention research, randomized controlled trials (RCT's) are considered the gold standard. In such trials, individuals (or clusters of individuals, such as classes or schools) are randomized into an intervention or control arm, and the prevalence of unhealthy behaviour is measured before and after the intervention. In TEENAGE, data from such studies will be stratified by indicators of SEP; as a rule of thumb, this will be done by dichotomizing the SEP-variable (high – low). If SEP indicators are continuous variables, the median will be taken, unless important other reasons will make another cut-off point preferable. In case of categorical variables, groups will be constructed of more or less equal sizes. This results in four groups: 1) low SEP and intervention arm (IL), 2) high SEP and intervention arm (IH), 3) low SEP and control arm (CL), and 4) high SEP and control arm (CH), with measurements before (T0) and after the intervention (T1).

In general, interventions selected in TEENAGE will be aimed at preventing increases in unhealthy behaviours that are still absent (e.g. smoking in young adolescence), at decreasing the prevalence of unhealthy behaviour in the general adolescent population (e.g. binge drinking) or at promoting healthy behaviours (fruit and vegetable consumption). Crucial for the reduction of socioeconomic inequalities in unhealthy behaviours – and therefore the focus of the statistical analyses in RCT' s – is that the difference in prevalence between the higher and the lower socioeconomic group in unhealthy behaviours after and before the intervention is reduced, e.g. becomes smaller in the intervention group than in the control group.

Some studies may exclusively concentrate on lower socioeconomic groups, which may be the case for interventions in lower vocational schools [42]. Such studies are eligible in TEENAGE. However, it needs to be recognised that the intervention may also affect individuals from higher socio-economic groups. Some studies, for example studies evaluating the introduction of a new policy, may only use pre- and post-measurements, leaving out a control group. Such studies can only result in conclusions about a reduction in socioeconomic inequalities in health-related behaviours under the assumption that socioeconomic differences in the general adolescent population for these behaviours do not widen or shrink. Currently, widening inequalities in health-related behaviours have been reported among US adolescents [19] and therefore studies with this design need to be interpreted carefully. Finally, some studies may have no baseline information, and are only able to provide information after the intervention. Such studies are only able to provide a statement about the reduction of socioeconomic inequalities in health-related behaviours under the assumption that socioeconomic differences before the intervention were the same in the intervention and control group. This can be the case for interventions with regard to smoking and alcohol consumption; in young adolescents the prevalence of smoking or alcohol consumption can still be about zero.

Analyses can be targeted at absolute socioeconomic differences (e.g. prevalence rates), but some studies may also have information about the relative socioeconomic differences. It needs to be considered that same absolute changes across socioeconomic groups may result in widening of inequalities. If possible, studies will therefore re-evaluate the interventions both in terms of absolute and relative socioeconomic inequalities.

Finally, many intervention studies may have been conducted in settings, in particular the school setting. It cannot be excluded that some of these studies – performed about ten years ago – have not been analysed according to current state-of-the art measures. In particular, multilevel modelling to account for clustering of data within higher than individual levels (classes, schools) may not have been applied, but will be applied if required in the re-analyses of the interventions [43].

### Transferability of evidence

There is considerable evidence of differences in the size of socioeconomic inequalities in health across Europe [44-46]. Suppose that an intervention were shown to be effective in reducing inequalities in health behaviors among adolescents in one specific country. Whereas this result may be of direct interest for the formulation of equity oriented health promotion in that particular country, the relevance for other countries is less immediate. If epidemiological and policy contexts strongly differ, it is doubtful whether application of an intervention to a new "target" country would be equally effective as in the "host" country where it was developed and evaluated. Further evaluation is needed before findings of intervention research can be transferred from one country to other countries, and before such findings can be assumed to have general applicability across Europe.

A main task of the TEENAGE project is to assess the transferability of findings of intervention studies from specific "source" countries to a number of "target" countries within Europe. This assessment will be made in a systematic way for different types of interventions applied to different health behaviours. The five "target" countries to be included will represent both the northern, southern, central and eastern parts of Europe. In each "target" country experts in health promotion will assess whether interventions with a shown ability to reduce inequalities in health behaviours elsewhere, will also be able to do so in their own country, and how effectiveness might be enhanced in the national context.

The transferability of findings depends on both epidemiological situation and the policy context. Epidemiological aspects will be evaluated in relationship to the health behaviour of interest. With regards to smoking, for example, European countries may strongly differ with regards to the phasing of the smoking epidemic and the magnitude of socioeconomic inequalities in smoking initiation [47,48], and possibly also with regards to the main determinants of smoking initiation among adolescents in different social classes. The policy context has to be assessed in relationship to this epidemiology and, in the case of smoking, include, the extent to which effective tobacco control policies have been implemented, the extent to which smoking initiation rates have been affected by these policies, and political support for further intensification of tobacco control at national and local levels.

In TEENAGE, a "transferability instrument" will be developed to support a systematic consideration of all relevant factors. Through the application of this instrument, health promotion scientists and policy makers can estimate the effectiveness of an intervention developed elsewhere, and they can identify opportunities for adoption to the national and local context in order to increase effectiveness. Finally, the application of this instrument to different interventions in different "host" countries will help in identifying the factors that most often facilitate or impede the cross-national transfer of findings from intervention studies.

### Initial search findings

The search procedure resulted in a number of studies for physical activity (n = 20), diet (n = 17), smoking (n = 21) and alcohol consumption (n = 4) (references of these studies are available at ). Table 3 shows that the majority of interventions used the school as a setting for the intervention. Some of these interventions only used the school to recruit individuals for an intervention targeted at individuals, e.g. to provide forms of health education. A substantial number of interventions used the school to recruit individuals for individually targeted interventions in combination with involving parents and/or friends. Only few interventions entirely focused on changing aspect of the school environment.

**Table 3:** Overview of types of interventions by setting obtained from an initial search in TEENAGE

Setting	Health education	Environment	Education + Environment	Laws, regulations	Health education + laws	Environment + law	Health education + environment + law
Individual	1 (PA)* 2 (A)	n.a.					
Home, family	2 (N)						
School	6 (PA) 4 (S) 5 (N)	1 (S) 1 (N)	9 (PA) 12 (S)** 1(A)^§^ 6 (N)		1 (S)	1 (N)	2 (N)
Neighbourhood/friends			1 (PA)^§§^				
National							
GP	3 PA 3 (S)^§§§^ 1 (A)						

*S = Smoking interventions, PA = physical activity interventions; N = nutritional interventions; A = alcohol interventions

** Two studies included a household component

*** 3 interventions included a family component and 3 interventions included a family and a community component

^§ ^including a peer component and a family component

^§§ ^including a school-based component

^§§§ ^one study in dental health clinic

## Discussion

Socioeconomic inequalities in health-related behaviours are well described [18], but evidence of effective interventions and policies to prevent these inequalities is currently limited. Scarce evidence of approaches to reduce socioeconomic inequalities in health mainly concentrates on the reduction of inequalities in adults. Project TEENAGE aims at extending knowledge by exploring possibilities for the prevention of socioeconomic inequalities in health-related behaviours in adolescence. It therefore adopts a pragmatic approach by synthesising evidence from intervention studies in the general adolescent population, after a stratified re-analyses by indicators of SEP.

A potential strength of TEENAGE is that it allows comparison of evidence of effectiveness within and across types of behaviours, as well as by setting. The approach will, in a cost effective and timely way produce directions for further development of health promotion strategies with a specific aim to reduce socioeconomic inequalities in the health-related behaviours. The project however, is also confronted with weaknesses in current research, perhaps the main weakness being that none of the studies selected will have been designed with the primary focus to explore differential effects across socioeconomic groups. As a result, indicators of SEP may be sub-optimal and studies may have limited power to detect intervention effects for specific groups. As a consequence, indicators of SEP often will need to be dichotomized instead of creating more groups and small groups will hamper the detection of significant interventions effects in both high and low socioeconomic groups. Conclusions and recommendations will therefore be mainly based on the direction of the difference in interventions across high and low socioeconomic groups in several studies, rather than on statistical significance. Another potential limitation of the project is the presumable inability to pay sufficient attention to aspects which may eventually reduce inequalities community-wide. This includes for example the recruitment of participants, the implementation of interventions, and the compliance among participants [49]. It cannot be excluded that these elements attenuate the community wide-effectiveness of efficacious interventions, particularly for lower socioeconomic groups. For example, particularly in the case of health education – including tailored interventions – selective participation cannot be excluded, whereby lower percentages of individuals from lower socioeconomic groups are included in the evaluation. Further, if those included are healthier than their non-participating peers from the same lower socioeconomic groups this would also influence the results, but only to the extent that this occurred more in the lower than in the higher socioeconomic groups.

According to the principles of a "planned approach" of health promotion, descriptions of socioeconomic inequalities in health-related behaviours should be followed by studies investigating the explanation of these inequalities, before interventions can be developed. Derived from evidence about the explanation of socioeconomic inequalities in health, one can speculate that most likely, a mechanism of social causation – where indicators of SEP are associated with health-related behaviours via intermediary factors – underlies the inequalities. According to social-ecological models [27,28], these intermediary factors can be intra-individual characteristics (such as attitudes, social norms and self-efficacy) as well as environmental characteristics at the household level, and the social and physical environmental level [36]. Thus, research is needed aimed at obtaining knowledge about the most relevant intermediary factors in the explanation of the inequalities. Such knowledge however, is still scarce and it may be expected to take a while before there is a robust understanding of the explanation of inequalities in health behaviours in adolescents. It is important to link information from such studies to the interventions identified in TEENAGE.

In 2008, the search strategy has been developed and interventions have been selected. Currently, a selected number of studies will be re-analysed in TEENAGE and the results of these re-analyses will be integrated in 2008. Interventions will be translated in guidelines for further research and policy recommendations in early 2009. The framework and the general rules for analyses also allow stratified analyses to be conducted in new or currently unidentified intervention studies. Such additional information can strengthen the findings of the project. In fact, given robust and substantial socioeconomic inequalities in health-related behaviours, it may be a matter of ethics to test if and how publicly funded interventions can contribute to the reduction of socioeconomic inequalities in health-related behaviours. All experiences together in TEENAGE will therefore result in the provision of guidelines for how to operationalize and test for differential effects across socioeconomic groups in future interventions. TEENAGE will concentrate on the equity dimension in health promotion among adolescents and as such, the project is linked with other important equity initiatives, for example in the field of conducting systematic reviews [50].

In conclusion, still little is known about strategies in health promotion in adolescents contributing to closing the gap in health between individuals from higher and lower socioeconomic groups. TEENAGE is among the first systematic scientific approaches across Europe to obtain such knowledge.

## Appendix 1: A detailed description of the search strategy

This document aims at describing the strategy to find existing interventions for re-analysis.

For all forms of behaviours, a systematic search strategy will be conducted based on the following procedure:

- Intervention studies which appeared in the international scientific literature since 1995, and conducted since 1990 are included; studies published before 1995 and/or interventions conducted before 1990 are excluded from the search;

- Only studies published in the scientific literature are included; grey literature is excluded from the search;

- Only studies in English language are included.

- Only studies conducted in Europe are included.

- Work package leaders adapt the general search strategy to the type of behaviour for which they are responsible.

The search will be conducted in the Pubmed database, and will be based on MESH terms. Specifically, the following MESH terms will be combined:

("Primary Prevention" [Mesh] OR "Health Education" [Mesh] OR "Preventive Health Services" [Mesh] OR "Risk Reduction Behavior" [Mesh] OR "Community Health Services" [Mesh] OR "Allied Health Personnel" [Mesh] OR "Health Personnel" [Mesh]) AND ("Adolescent" [Mesh] OR "Adolescent Behavior" [Mesh]) OR "Child Behavior" [Mesh]) AND ("Intervention Studies" [Mesh] OR "Randomized Controlled Trial " [Publication Type] OR "Clinical Trial " [Publication Type] OR "Evaluation Studies as Topic" [Mesh] OR "Evaluation Studies " [Publication Type])

## Competing interests

The authors declare that they have no competing interests.

## Authors' contributions

FJvL initiated the project and drafted the manuscript. IdB, KIK, NL, LM, FF, AEK and JPM contributed to the initiation of the project, are work packages leaders within the project, and commented on the earlier drafts of the paper. All authors read and approved the final manuscript.

## Pre-publication history

The pre-publication history for this paper can be accessed here:


